# Nutrition in Lithæmia

**Published:** 1886-02

**Authors:** Chas. G. Stockton

**Affiliations:** Professor Materia Medica, Niagara University; 371 Porter Avenue, Buffalo, N. Y.


THE BUFFALO Medical AND Surgical Journal

VOL. XXVI. FEBRUARY, 1886. NO. 7.

### Original Communications.

### NUTRITION IN LITHÆMIA.

By CHAS. G. STOCKTON, M. D.

Professor Materia Medica, Niagara University.

Lithæmia is called the gouty dyscrasia, but it is so unlike gout that I think it might as well stand as simply a dyscrasia.

Gout is rare; lithæmia is common.

Gout comes unexpectedly, like a robber at night, and, after a while, leaves you feeling better than before, thankful that life was spared, and with faculties awake to regain what has been stolen.

Lithæmia, like a mendicant, is always at your door; one of the well-dressed kind that follows you to your table, to your chamber, to your study. The only relief is to give, and keep giving; and, truly, 'tis more blessed to give than to receive.

Gout may be driven off with drugs, but lithæmia flourishes thereon.

Rheumatism is as near to gout as is lithæmia. They are related to each other, but are not joined in holy wedlock.

I appreciate the difficulty of defining such disorders, and confining them to their definitions, but I wish that writers would separate them a little more.

Lithæmia implies the presence of a certain irritant, lithic acid, within the blood; and, for the present, granting this, the question is, first, how did it get there? second, how shall we get it away?

Is it from fault in the *prima via?* or from an overworked liver? or too little oxygen? or from disturbed innervation? or—what?

That there is a condition called biliousness by some, frequently met with by all, and having a train of manifestations that are greatly modified by the character of the food taken, there can be no doubt. The disease—if disease it may be called—is generally attributed to indigestion by weakened, dilapidated digestive organs—the stomach, intestines, liver and pancreas.

Nevertheless, we are continually finding people who seem to have sound organs and no dyspepsia, and yet are "bilious." I venture the prediction that if the digestive organs of these people could be subjected to scrutiny, they would be found without fault. The aliment is wholesome; the primary digestion is complete; into the fluids of the body are poured the chyme and the chyle, properly prepared.

The sanguinous system is freighted with nutrition for distant provinces, and its transportation is hastened in direct ratio with the heart's action, and the heart's action is effectual or ineffectual, in proportion as this burden of nutrition is promptly received and disposed of at its myriad destinations, the cells, the protoplasm of all the body. The actual nutrition is cellular. The question is not how much the blood will receive, but how much the cells will assimilate. Hence, we talk of tissue metamorphosis, and of the proper oxygenation of the blood, well knowing that in the healthy body the matter of waste depends upon oxidation, and that the matter of repair depends upon the appropriation—adaptation of nutriment from the fluids of the body by the cells of the organs.

The blood has two hands. In one, the plasma, it conveys nutriment to the cells; in the other, the hæmaglobine, it carries oxygen to them, energizing and, through activity, depleting them, rendering nutrition possible.

We have reached a cardinal point.

Tell me why some healthy animals are "easy keepers" and others "hard?" Tell me why often small eaters grow fat and great eaters remain lean? And tell me why, when age creeps on apace, that some grow pursy and others shrivel up? *

Such examples of development and decay are common and are hereditary—"like father, like son"—and, when we say this tendency is hereditary, we say that it is vital. In the scheme, in the architectural design of the being, are to be found the answers to these questions; and there, also, and somewhat likewise, will be explained why the tissues of some are prone to nourish and waste properly and in order, while others act out of time and out of tune.

While the secrets of vital phenomena are unfathomable, some of their manifestations we may sound.

The nervous system is intimately connected with the whole process of nutrition. Tickle one, and the other always laughs.

A nervous system distraught by venery or the loss of sleep, by malaria or the taking of drugs, is attended by waste. This is partly through the vaso-motor system and arterial tension so prolonged that there results a partial venous stasis—cyanosis, and the tissues are poorly fed. The effete material is not carried out of the body as rapidly as it should be, and besides that, what I think of very great importance, the lymphatic circulation is hindered. Let us remember that the lymph spaces and the terminal blood vessels cohabit; that there is a constant interchange of substance between them; that blood supply and blood pressure have a mighty deal to do with the motion of the lymph current, just as the quality of the blood has to do with the qual-

*Surgical Pathology (Paget), pages 82 and 83.

ity of the lymph. Let us also remember that the blood is dependent upon the lymphatic system for its corpuscles, so that oxygenation, to be perfect, must have an active lacteal tract; that the real nutrition of tissue cells is from the lymph in which they bathe; that the rapid abstraction of lymph from animals proves more speedily fatal than hemorrhage; and we must realize how important in this question are the lymphatics.

With the nervous, the sanguinous, the lymphatic systems in united harmonious activity, the tissues would be properly fed and biliousness probably forgotten. But, with these perturbed, there is imperfect metabolism, suboxidation and lithæmia.

The tissues take up less nutrient material from the blood, and that fluid soon becomes overcharged both with that which is effete and that which is not. The nerve centers are further distracted, among others those which preside over the circulation, resulting, in numerous instances, in still higher arterial tension, associated with a slow pulse, greater venous and lymphatic engorgement, oxaluria, and a multitude of symptoms that are as hangers-on.

This state of affairs must affect the liver unfavorably, retard the portal circulation, and offend the primary digestion.

When there is a solidified lung, there is a backing up of venous blood; and this illustrates the somewhat similar fact that when the cells are indolent and the tissue changes slow, there is a backing up of nutrition, if I may be pardoned the solecism.

The liver is now a sluggard, and occupying an intermediate position between the primary digestion and the ultimate nutrition, is capable of deranging both.

Not to be too elementary, it is enough to say, experience teaches that when the liver is thus disordered, improvement follows, lessening the work of that organ.

This may be done, first, by limiting the amount of food; second, by permitting only such articles of diet as the liver receives kindly; third, by making the primary digestion as complete as possible.

The first is easily accomplished, where one can overcome that pernicious habit of stuffing patients, and for which the profession is to blame.

Study of the second point has led some to advise a dietary which admits of proteids, but excludes starches, sugar and alcohol; others, however, allow farinaceous food, but limit the eating of azotized substances.

It seems to me that these contradictory views may be satisfied by studying the third point, the perfection of primary digestion.

The proteids which leave the duodenum as peptones, in some manner changed by absorption, enter the portal circulation as a form of albumen. This, in the liver, meets with unknown changes. Some is transformed into glycogen, a carbo-hydrate. Much albumen, in some way modified by its liver experience, passes on into the circulation to be consumed by the cells, and it would be expected that the resulting urea and uric acid would prove most pernicious to those suffering from acute gout. So far as I know, the experience of all the world confirms these expectations, except that of Dr. Draper and his adherents. But we are indebted to Dr. Draper for pointing out that a nitrogenous diet is, of all, most agreeable to some lithæmics, so-called. That it is the best diet for all lithæmics, I do not believe, and for the reasons that follow:

Some bilious individuals have good stomachs, but have weakened intestinal digestion. They digest starch with difficulty, but meat with comfort; and in such cases meat will be found to agree with the liver. Other bilious individuals, with gastric catarrh, abhor albumenoids; and when such substances are eaten, they are poorly peptonized and, I suggest, are absorbed into the portal vein, improperly prepared for their liver tenement, and reach that organ as disturbers of the peace. At such a time, a man may have auto-inoculation, whether from ptomaines or other poisons even less known.

Does anyone say that this is impossible? Then, upon him rests the burden of telling *why* it is so.

Of one thing I am sure: that it is, in a proportion of cases, impracticable to continue the azotized dietary, for the reason that your patient will grow worse upon it; but when you put him upon a diet of starches and fats, properly re-enforced with diastase and pancreatin, there will ensue a great betterment.

Do not let me overstate myself. The starchy diet is often the very worst, and the albumenoid the best. This is so for the reason that the primary digestion does not change the starch into a suited glucose. Nevertheless, it is absorbed by the *vena porta*, together with irritating acids, and instead of a transformation into glycogen, and from glycogen back again into a purified, chastened glucose, adapted to its new life in the better world of the general circulation, where it and its companion, oxygen, shall serve the vital cell, it proves a defiler in the house of its father. It deranges the liver's glycogenic function, and part of it slops over, so to speak, and enters the blood as a doer of evil.

If asked how I would manage lithæmia, I would answer: avoid quinine; look carefully after gastro-intestinal catarrh; keep open all the sewers of the body, including the liver; be most mindful of the primary digestion and suit the diet to the case; using, if needed, pepsin and hydrochloric acid with albumenoids, vegetable diastase with starches, and extract of pancreas with milk and the fats, after the admirable plan of Roberts, of Manchester. And finally, the most important and most neglected agent of all, oxygen, should be gotten into the body. Do this by massage, which will improve the capillary circulation and hasten the lymph stream; by inhalation of oxygen, which promises much in this direction; by change of climate, or, if the case is not urgent, enjoin, as far as practicable, an active out-of door life, with horse-back riding; for if there is anything that is truly anti-lithæmic, exercising, exhilarating, tingling a man into the belief that he is related to the gods, that thing is equination.

371 Porter Avenue, Buffalo, N. Y.

